# Behavioral activation for depression in groups embedded in psychosomatic rehabilitation inpatient treatment: a quasi-randomized controlled study

**DOI:** 10.3389/fpsyt.2024.1229380

**Published:** 2024-04-25

**Authors:** Ursula Melicherova, Tobias Schott, Volker Köllner, Jürgen Hoyer

**Affiliations:** ^1^ Psychosomatic Rehabilitation Research Group, Department of Psychosomatic Medicine, Center for Internal Medicine and Dermatology, Charité – Universitätsmedizin Berlin, Berlin, Germany; ^2^ Institute of Clinical Psychology and Psychotherapy, Technische Universität Dresden, Dresden, Germany; ^3^ Department of Psychosomatic Medicine, Rehabilitation Center Seehof, Federal German Pension Agency Teltow, Teltow, Germany

**Keywords:** behavioral activation, cognitive-behavioral therapy, group therapy, inpatient setting, psychosomatic rehabilitation, international classification of functioning, depression

## Abstract

**Background:**

Behavioral activation (BA) and cognitive-behavioral therapy (CBT) have shown to be efficacious treatment methods for depression. Previous studies focused mostly on the outpatient treatment either in group or individual setting. The present study aimed at comparing the efficacy of group treatment BA *vs*. CBT, when embedded in inpatient psychosomatic rehabilitation treatment.

**Methods:**

375 inpatients were randomly assigned to either BA (N = 174) or CBT (N = 201). We used established scales for depression such as the Beck Depression Inventory II (BDI-II, self-rating), the Quick Inventory of Depressive Symptomatology (QIDS; expert rating) and the Behavioral Activation for Depression Scale (BADS) to assess changes over the course of the treatment and at follow-up (4 to 6 months). In addition, we measured disability-related functioning with the Mini-ICF-APP, a rating scale built in reference to the International Classification of Functioning, Disability and Health (ICF). Multilevel models with repeated measures were conducted to examine the differences between groups in relation to change over time with patients’ random effects.

**Results:**

Both group formats showed substantial reduction in depressive symptoms at the end of treatment (d= 0.83 BA *vs*. d= 1.08 CBT; BDI-II) and at follow-up after 4 to 6 months (d = 0.97 BA *vs*. d = 1.33 CBT, BDI-II; and d = 1.17 BA *vs*. d = 1.09 CBT, QIDS). There were no significant differences between treatment approaches. At least 50% symptom reduction was achieved by 53.7% and 54.2% in BA *vs*. CBT respectively. Reported activation levels increased from pre- to posttreatment (d = 0.76 BA *vs*. d = 0.70CBT), while showing loss of increment between the end of the treatment until follow up in both formats (d = 0.28 BA *vs*. d = 0.29 CBT).

**Discussion:**

Both modalities led to significant improvement of symptomatology and functioning at the end of the treatment and at follow-up, thus for the first time demonstrating the practicability of BA in rehabilitation clinics. Considering its lower requirements regarding cognitive abilities and its easier implementation, BA proved to be a good alternative to other psychotherapeutic treatments.

## 1 Introduction

Affective disorders are one of the three leading non-fatal health burdens across the globe (1). They are not only common and costly (e.g.; due to *premature retirement*), but also associated with considerable morbidity and mortality. As effective treatments are available (2) it is primarily the implementation of effective as well as efficient treatment methods for these disorders which poses a major challenge for service provision worldwide. Accordingly, there are continuing efforts to disseminate efficacious treatments into the diverse segments of health services. Furthermore, considering patient preferences it is important to make different types of efficacious treatments available to establish a more precise matching between individual patients and individual psychotherapies (3).

Psychotherapy has been found to be at least as efficacious as pharmacotherapy in mild to moderate depression (4) and many psychotherapeutic interventions could demonstrate their long-term efficacy (3). Two types of psychotherapy are exceptionally well-researched and have proven their efficacy based on many randomized and controlled studies: cognitive-behavioral therapy (CBT) and behavioral activation (BA). Despite their demonstrated and comparable efficacy (5) one *advantage* of BA might be that it is easier to understand (for patients) and, hence, to implement (into service structures): Cognitive-behavioral therapy for depression includes different components focusing both on behavioral as well as cognitive change, which at least some patients may find complex and time-consuming (6). Particularly its cognitive components can be more difficult to understand for marginalized populations (e.g., due to language, cognitive or economical barriers). On the other hand, there is strong evidence that a simple behavioral approach, behavioral activation (BA), is easy to administer (e.g., 7) and efficacious within many diverse patient populations (e.g., 8–10). Dimidjan et al. (11) showed in an RCT a significant amount of extreme non-responders in the CBT study arm but not in the BA. Furthermore, Jahoda et al. (12) conducted a RCT with intellectual disabilities who showed a substantial decline in depressive symptoms after BA treatment. The main focus of BA lies on activity monitoring and activity planning (13), which both aim at directly counteracting depressogenic factors such as lack of reinforcement, extinction of instrumental behavior, and excesses of aversive control. Recent research has shown that brief activity planning instructions alone can have relevant effects for emotion regulation and prevention (14). Given the clear focus and the relative simplicity of BA, we assume that it can be a viable alternative to CBT in many settings, especially when large groups of patients need to be treated in relatively condensed ways, as is the case in inpatient psychosomatic rehabilitative treatment (15).

The positive effects of CBT and BA mentioned above seem to be observable across different modalities such as individual *vs*. group treatment (16, 17). Group psychotherapy in particular constitutes a competing alternative to individual psychotherapy as it makes psychotherapy accessible for many patients and is clearly more economical (18). Furthermore, it is the most viable form of psychotherapy in inpatient settings, and in countries such as the UK, the USA, and Germany, group psychotherapy is the most prevalent modality of inpatient psychological care (19–21).However, as countries such as the USA are following the trend of outsourcing psychological treatment away from psychiatric wards, there is less data on inpatient than on outpatient treatment (22). This is a shortcoming of high clinical relevance, as it has been shown that inpatients do not represent the same target population as outpatients as they demonstrate more severe symptomatology, are often chronically ill, and diagnosed with higher number of comorbidities (22).Hence, the effects observed in outpatient settings may or may not be valid in inpatient contexts as well. The need of independent research on that subject, especially regarding group therapy, is obvious.

Another research requirement is the more thorough study not just of patients’ symptom reduction but also of their improvement in quality of life, functioning and participation within society (23). In order to do so, clinical research as well as clinical decision-making regarding mental health disorders must consider level and pattern of functioning (24). As a system for providing data on disability-related functioning and non-fatal health outcomes, the World Health Organization (WHO) proposed the International Classification of Functioning, Disability and Health (ICF, 25). ICF acts complementary to ICD (see ICF, 2001) and will be consistently applied in this study. The terms ICF, functioning, and disability-related functioning and non-fatal health outcomes will be used interchangeably.

Summarizing, the aim of the present study was to compare BA to cognitive-behavioral therapy, embedded in a group inpatient treatment of depressed patients with a high number of comorbidities. Firstly, we expect patients in both treatment modalities to reduce depressive symptoms with BA exhibiting a significantly larger reduction. Furthermore, we expect patients with lower education level to show steeper decline than those with higher one. Secondly, we expect both groups BA and CBT to increase in activation, especially patients with lower education level in the BA group. Thirdly, participation/impairment: We expect both groups BA and CBT to show a decrease in social and work impairment.

Given the context of a multidimensional inpatient treatment for chronic mental disorders, which focuses on life and work participation, we also examine the changes in terms of the ICF (25).

The study was conducted in the naturalistic setting of two psychosomatic rehabilitation clinics but based on the admission date of the individual patient, quasi-randomization of patients to BA *vs*. CBT could be established.

## 2 Materials and methods

### 2.1 Study overview

Psychosomatic rehabilitation is a specific treatment setting within the German health provision system. It is closely related to the ICF hence it must encompass certain treatments: treatment of symptoms (psychotherapy and/or medication), training of capacities (e.g., assertiveness, social competence), change of context, social support (e.g., pension, other monetary services), salutotherapy. The average stay is 5 weeks with the option of prolonging the stay by up to 2 weeks. All patients get individual (1x 60 min/week) and group psychotherapy (2 x 90 min special group therapy e.g., depression; 1 x 90 min interpersonal group), occupational therapy, muscular and balance training (2 x week), and aerobic exercise (2 x week). If deemed necessary, patients can also participate in occupation related either individual or group counseling. For more information on the German psychosomatic rehabilitation system and its relatedness to the ICF see Linden (19).

Within the above-described setting, we systematically varied the specific group psychotherapy component (see below 2.4 randomization), which was the main psychotherapeutic ingredient of the treatment. Groups conducted according to a BA manual (see below) with those conducted according to a standard CBT manual (see below) were compared.

### 2.2 Setting

Patient acquisition and treatment took place in two inpatient psychosomatic rehabilitation hospitals (Seehof in Teltow, and Clinic Bavaria in Kreischa, both in Germany). Both belong to a larger network of hospitals run by the German Pension Insurance (19). Access is open to the public and costs are covered either by the German Pension Insurance or by the general healthcare system in Germany. Due to differences in the interpretation of federal state requirements on public hospitals by hospital CEOs, one methodological difference needs to be mentioned. Group sessions could only be videotaped in hospital 1, whereas in hospital 2 adherence was checked in regular supervisions. Furthermore, different hospital policies and organizational processes, stuff shortages during the COVID-19 pandemic led to following differences: the Mini-ICF-APP was conducted as an external rating at T0 and T3 in hospital 1 and self-rating at T4. In hospital 2, Mini-ICF APP was only conducted as self-rating at T0, T3, T4. Furthermore, QIDS-C was conducted at T0 and T4 at the hospital 1, and at T0, T3 and T4 at hospital 2.

### 2.3 Patients

Adults undergoing psychosomatic rehabilitation between February 2019 and November 2020 with a diagnosis of a depressive syndrome (assessed with M.I.N.I. and QIDS, 26, 27) were recruited into the study (N= 375, women= 76%). Additionally, 56% of the patients in BA and 63% in TAU also showed particularly problematic occupational problems at admission, assessed via SIMBO-C (28). A total score of SIMBO-C (based on 6 scales: age, motivation/expectation, subjective occupational prognosis, socio-medical problems, health-related occupational impairments) was used to assess whether additional occupation counselling was needed. Exclusion criteria were a history of or current psychotic symptoms, substance dependence or abuse, or a severe comorbid anxiety disorder with pronounced avoiding behavior. Also, patients with organic brain disorders and/or severe untreated sleeping apnea were excluded from the study. All participants provided oral and written consent to be included in the study. Patients were blinded to the purpose and study hypothesis. Informed consent included the information that participants would participate in a treatment condition expected to offer an effective treatment for depression.

### 2.4 Randomization

All patients considered eligible for the study purposes were randomized into either BA or CBT The randomization was conducted as following: The study nurse responsible for the general coordination of all treatments conducted the randomization. The study nurse was blinded for the purposes of the study as well as the content of group modalities. Upon inclusion in the study, patients were assigned to the category “the group therapy for depression” in the computer system of the hospital. From this group, the nurse then assigned them either to BA or CBT based on the odd or even number of the week.

### 2.5 Procedure and treatments

Data was collected from February 2019 until September 2020 under the project title “Behavioral activation for depressive syndromes in rehabilitation”. The study was approved by the local ethics committee of the Technische Universität Dresden (Germany; EK 327082018) and preregistered at the German Clinical Trials Register (DRKS 00016495). Patients were randomly assigned either to a behavioral activation group (BA, N = 174) or a cognitive-behavioral group-psychotherapy (CBT, N = 201) for depressive syndromes. Based on clinical expertise of the medical director or assistant medical director, if deemed inevitable for the achievement of therapy goals, the stay was prolonged by up to two weeks. In order to fulfill the rehabilitation-guidelines (29) of the German Pension Insurance Union, group therapy is mandatory throughout the whole stay. Therefore, patients attended a psychological group treatment with the focus on relaxation/mindfulness exercises and problem-solving exercises after the prolongation of their stay. CBT group therapy format adhered to the well-established and scientifically evaluated methods of the cognitive-behavioral therapy manual by Hautzinger (30). The group therapy comprised psychoeducation, cognitive therapy, session(s) on social skills, and (restricted to one session) behavioral activation with pleasant activities. The behavioral activation (BA) group was based on the manual on behavioral activation in groups by Hoyer and Vogel (31), which focuses strongly on value-based activity monitoring and planning. Sessions took place twice a week, each lasting 90 min. In total, 75 groups (each consisting of 3-12 patients) were analyzed.

### 2.6 Adherence check

All sessions of hospital 1 were videotaped, and the adherence was checked by regular supervision. In addition, we conducted a formal analysis of the adherence with the respective treatment manual. For this purpose, the rating scale AVADIR (32) was developed. The scale was created using a rational-deductive approach from various instruments evaluated in practice to assess manual adherence for the two treatment conditions (BA *vs*. CBT). AVADIR is a 64-items 7-point Likert scale with four subscales: *behavioral activation techniques*, *cognitive techniques*, *generic group-psychotherapeutic techniques*, and *extraneous techniques.* Based on these items, a total sum score is calculated. In addition, it is possible to map a differentiated representation of individual treatment techniques in the context of the therapy sessions to be evaluated. Each video included in the analysis was rated by 3 trained members of the study, and interrater reliability was calculated. The adherence in hospital 2 was provided by regular supervision meetings.

### 2.7 Assessment plan and instruments

Diagnoses were established according to the following procedures:

All patients that were checked for eligibility in the study participated in a diagnostic interview, which was conducted by trained interviewers. Training was comprised of 3 components: a) shadowing of project coordinator (a clinical psychologist in an advanced psychotherapeutic training), b) participation in an online training on QIDS, c) performance of the interview in front of the project coordinator. The diagnostic instruments used in this study were the Quick Inventory of Depressive Symptomatology (QIDS, 27) and the Mini-International Neuropsychiatric Interview (M.I.N.I., 26). Patients were considered eligible when they reached minimum 8 points in QIDS and were screened for psychosis, alcohol abuse, and bipolar disorder with the M.I.N.I.

#### 2.7.1 Primary outcomes

Depressive symptoms’ severity was measured via the Beck Depression Inventory (BDI-II, 33). The BDI-II is a widely used 21-item self-report inventory measuring severity of depressive symptoms. Activation was measured with the Behavioral Activation for Depression Scale (BADS), via a 25-item (T0, T3, T4) (34) and 9-item (T2, T3) form (35). Additionally, QIDS-C scores were analyzed at Pre and T3 (hospital 2) *vs*. T4/Follow up (both hospitals).

#### 2.7.2 Secondary outcomes

**Figure 1:**
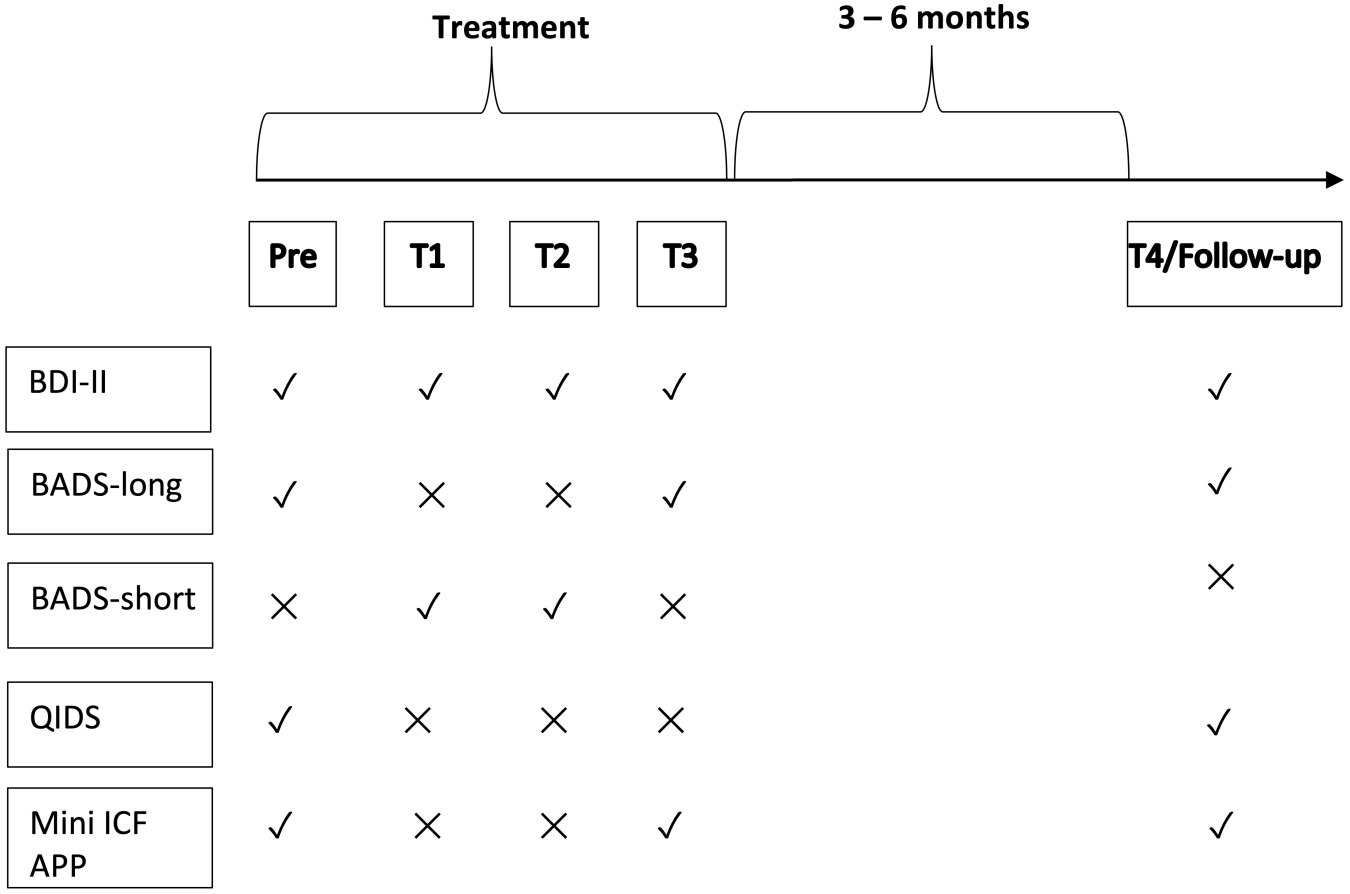
Study timeline. The figure depicts the order in which the questionnaires were administered to the patients. Time point Pre took place prior to the beginning of the treatment. T1, T2, and T3 were administered in the subsequent weeks. Follow up took place at 3 to 6 months. Check marks indicate that the questionnaire was administered and crosses that it was not. Due to differences in organizational processes, only in one hospital QIDS-C was conducted at T3. We did not include this data in the main analysis.

Disability related functioning was measured via Mini-ICF-APP (Mini-ICF-Rating für Aktivitäts- und Partizipationsstörungen bei psychischen Erkrankungen/Mini-ICF-Rating of impairment in activation and participation for mental illnesses) which measures either as a 13-item self-report (36) or 13-item external rating (37) following the 13 areas of ICF: adherence to regulations, planning and structuring of tasks, flexibility, applying expertise, competence to judge and decide, endurance, assertiveness, contact with others, group integration, intimate relationships, non-work activities, self-care, and mobility. Depression and activation scores were measured prior to the treatment and then in the 2^nd^, 3^rd^ and 4^th^ week of the treatment (see **Figure 1**), before the respective group session started (Pre, T1, T2, T3).

Follow-up took place at 3 to 6 months after the discharge from the hospital (T4). Both clinics followed the same procedure: 3 up to 6 months after the discharge from the clinic, patients were contacted by the trained study team via telephone. After oral consent was given, qualitative interviews concerning symptoms and work status, as well as the QIDS-C were conducted. Patients were then asked to fill BDI-II and BADS online, in pen-and-pencil form, or directly on the telephone, as has been done similarly by Senior, Kunik (38). Patients who did not complete the questionnaires received a maximum of five reminder phone calls (pen-and-pencil) or emails (online) after one and two weeks.

### 2.8 Therapists

A total number of 35 therapists (hospital 1, n= 25) were recruited to participate in the study. All therapists were either clinical psychologists (holding a master’s degree in psychology) or physicians (specialization in psychosomatic medicine), either fully approved in psychotherapy (CBT) or in an advanced postgraduate training in CBT. The training for both psychologists and for physicians specializing in psychosomatic medicine and psychotherapy are equivalent in Germany. Accumulated over both hospitals the mean clinical experience was 4.98 years (*SD*= *5.34)*, mean age 36.91 years (*SD*= 5.9). In hospital 1 68% were women *vs*. 90% in hospital 2. All therapists had participated in the workshop on CBT specific techniques for groups that are required for the psychotherapeutic training. In particular, as CBT is an integral component of the psychotherapeutic curriculum, all therapists took part in it. In addition, all therapists also participated in a specific training for the application of the manual for the study. The latter comprised of two lectures (with an average duration of 3 hours) that introduced the theory and specific techniques of the modality to which they were allocated. The allocation was not random as both therapists’ and hospital schedule had to be accounted for. Throughout the duration of the study regular supervision was available. In a nutshell, all therapists had received basic training in CBT prior to the study. However, only BA therapists received training in BA. CBT therapists also attended additional workshop on CBT right before they were allocated to this treatment modality.

### 2.9 Dropouts

**Figure 2:**
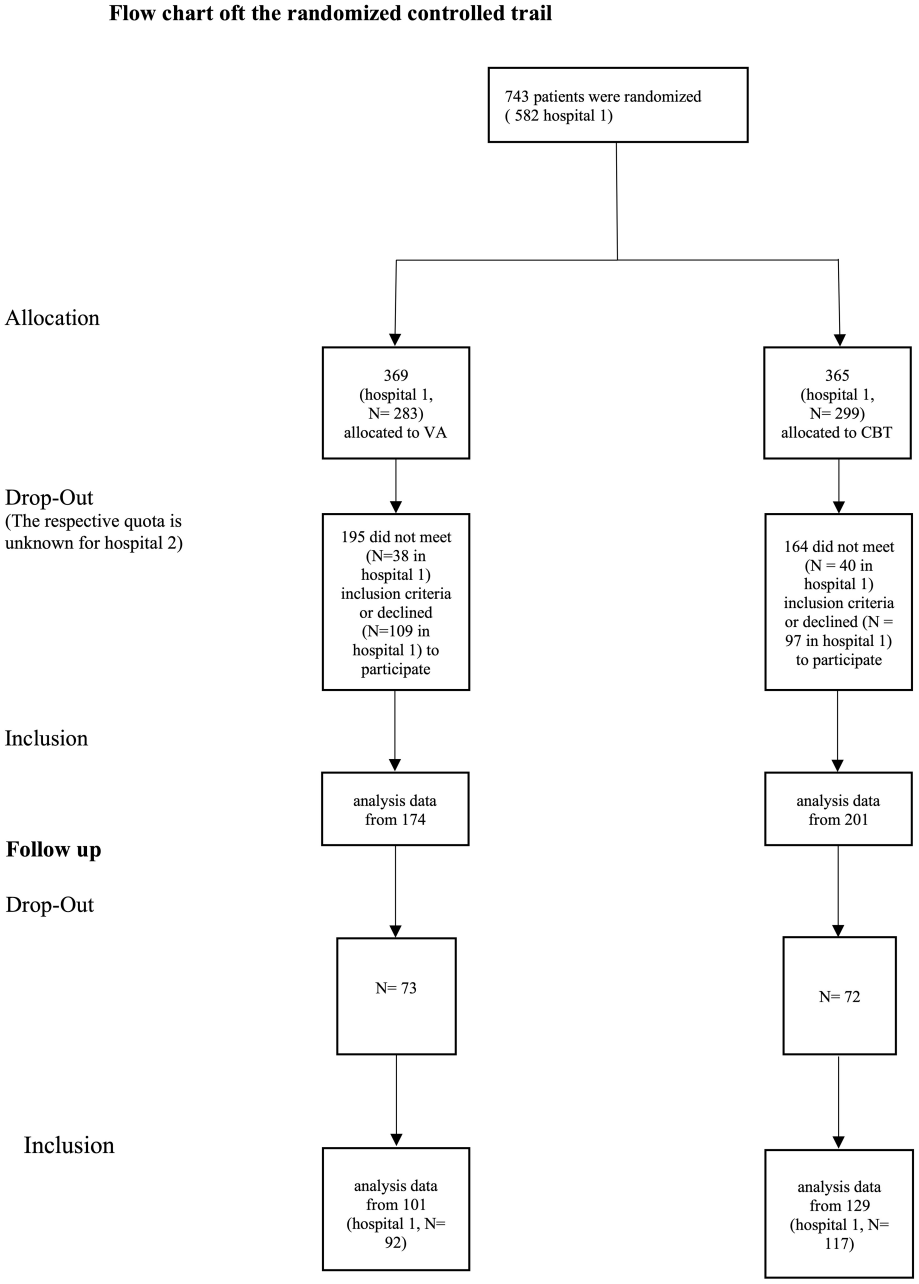
Sample flow chart.

**Figure 2** shows the study flow chart. Due to high dropout rates, especially at follow up, multilevel models were carried out as intent-to-treat analysis.

### 2.10 Statistics

Based on the combined assumption that BA might only be marginally more efficacious in typical patients (d = .20) but more efficacious to a moderate degree in those being less well educated (d = .50) and that both groups will constitute about 50% of the sample, we deduced a potential effect of d = .35. In addition, we corrected the *a priori* sample size for potential cluster effects following the proposals by Rutterford, Copas and Eldridge (2015) and which resulted in *a priori* sample size N= 500 (250 in each arm).

Due to lockdown during the first two COVID-19-waves, group treatment was unexpectedly cancelled which led to a significantly smaller sample size (see flow chart). Since the estimated sample size was not met, we interpreted the results based on the confidence intervals (CI, see **Supplementary Figure 1**) as described elsewhere (39, 40). CIs are a more sound alternative to *post-hoc* power analysis, which has been criticized for only giving tautological and non-informative results as all studies with non-significant results will always show low power (41).

Both outcome variables have a hierarchical structure in which BDI-II and BADS (scales: activation, avoidance, social and work impairment) responses (level 1) are nested within participants (level 2), who are nested within groups/therapists (level 3) and within hospitals (level 4). Mixed effect models (also called multilevel models or hierarchical linear models) are a more appropriate method than conventional unilevel analysis for such nested data (42). We used a mixed effects models approach with full information maximum likelihood estimation. Furthermore, mixed effect models are able to accommodate missing data and integrate time-varying factors (43). In particular, linear mixed models are also robust to violations of distributional assumptions (42). We report the results according to Luo et al. (44).

Data preparation and statistical analyses were carried out with RStudio (Version 1.3.109, RRID : SCR_000432) with R 4.0.3 and diverse additional packages available under: the reserved DOI: 10.6084/m9.figshare.22815773. Prior to the analysis, the differences in demographic variables between the group formats were tested via non parametric tests (Pearson’s Chi-squared test, Wilcoxon rank sum test or Fisher’s exact test), see **Table 1**.

**Table 1:** Selected socio-demographic characteristics of both treatment samples.

Characteristic	BA, N = 174*^1^*	TAU, N = 201*^1^*	p-value*^2^*
SIMBO-C value length of stay	55% 40	63% 41	0.121
*sex*			0.7
women	135 (78%)	152 (76%)	
*age*	54 (47, 58)	53 (48, 59)	0.5
*education level*			0.4
special needs school	0 (0%)	0 (0%)	
secondary school certificate	6 (3.4%)	8 (4.0%)	
compl.vocational training	98 (56%)	129 (64%)	
A-levels	45 (26%)	44 (22%)	
University degree	25 (14%)	20 (10.0%)	
ICD-Diagnosis			
f1 (history)	8 (4.6%)	10 (5.0%)	0.9
f40-f41	102 (59%)	102 (51%)	0.14
missings	0	1	
f42	9 (5.2%)	8 (4.0%)	0.6
F43.1	20 (11%)	17 (8.5%)	0.3
f5	4 (2.3%)	3 (1.5%)	0.7
F34.1	35 (20%)	38 (19%)	0.7
missings	1	0	

^1^ n (%); Median (IQR).

^2^ Pearson’s Chi-squared test; Wilcoxon rank sum test; Fisher’s exact test.

BA, behavioral activation; TAU, treatment as usual.

Simbo value= % of persons needing counseling regarding ICF.

To model the development of BDI-II scores between the two group therapy formats, a linear multi-level model was used. To analyze the BADS, the following steps were conducted. Scales *activation, avoidance, social and work impairment* were calculated with the *scoreItems* function from the psych package. See vignette of the package for more information. Each scale was quantified as a sum of items of BADS-Scales for each patient. For the BDI-II we used the total scores as outcome variable. Data was analyzed as a function of *treatment*, *time*, and *education level* as fixed effects and the interaction term was *treatment, time* and *education level*. To incorporate the dependency among observations within a person and a hospital, random intercept person nested within a *location* (hospital) were specified. We used the log likelihood test and the AIC criterion to determine whether the inclusion of each term improved the model fit, and the function *cohen.d* from the package effsize to calculate Cohen’s d effect size (45). Also, *QIDS* at follow-up scores were analyzed as a function of *baseline score, treatment* and *education level* via multilevel models. Random intercepts of groups nested within a location were included, too. Further details and R code are available in the **Supplementary Materials**.

Secondary outcome measure: As Mini-ICF-APP consists of ordinal data, one sample Wilcoxon signed-rank tests were conducted for each scale. Furthermore, due to high number of zeros in data set causing non-normal distribution, zero-inflation Poisson regression was conducted with interaction term Time x Treatment (46). Therefore, the Cohen´s d could not be calculated. The effect size (ES) r is calculated as Z statistic divided by square root of the sample size (47). Due to differences in data collection between the two hospitals, only one hospital was able to provide a clinician-administered scale rather than a self-rating, which were analyzed. The descriptive statistics of the self-rating from the second hospital are available in the **Supplementary Materials**.

## 3 Results

### 3.1 Descriptive statistics of the sample

The mean age in the study was 51.92 years (SD=8.65); 76% were women. For a comparison between the treatment groups see **Table 1**. In the present sample, most of the patients completed vocational training. All patients were diagnosed with depressive disorders and 66% were diagnosed with a comorbid mental disorder (see **Table 1**). Comparison of the socio-demographic variables did not reveal any significant differences between completers *vs*. non-completers of follow-up (see **Supplementary Table 1 in Supplementary Materials**).

### 3.2 Manual adherence

8% of the video tapes were of sufficient recording quality and could be selected for adherence checks according to the AVADIR scale. The adherence check was conducted after 13.5 months (out of 24 months). At this time in the hospital 1, 448 group therapy sessions were conducted (56 group therapies conducted x 8 sessions). Of the 448 group therapy sessions conducted, video excerpts of at least 30 minutes were available for 218 sessions. According to Dennhag, Gibbons (48), at least three group therapy sessions of six sessions each should be assessed per therapist to provide sufficient assessment of adherence. For a total of 15 therapists (BA=7; CBT= 8) at least one complete group therapy (8 sessions per run) was available. 22 complete group therapies (56%) were led by more than one therapist, so that these had to be excluded from analysis.

From this sample, a random sample was drawn from three different strata: 1) one of the three group therapies per therapist per condition (BA; CBT), 2) session number per group therapy (one from session 1-4 and one from session 5-8), and 3) session phase (beginning, middle, or end of session). Thirty-six therapy excerpts (duration: 30 minutes; BA: 18; CBT: 18) from each of six therapists (BA: 3;CBT: 3) were included in the analysis.

The overall adherence to the group manuals was measured via the total score. No differences were found between the therapists of both groups (BA: M= 3.94, CBT: M=3.57). The results indicate an average adherence based on the 7-point Likert scale of the AVADIR. The inter-rater-reliability was average (ICC_5_= .46 (95% K [.07,.71], p = .013). Furthermore, rated video segments conducted by BA therapists contained 66% behavioral techniques (M = 9.80, SD = 5.98); 34% generic group techniques (5.02, SD = 3.37) and 1% cognitive ones (M = 0.13, SD = 0.38). Rated video segments conducted by CBT therapists were found to contain 66% cognitive (M = 7.89, SD = 5.81), 22% generic (M = 2.65, SD = 2.40), and 12% behavioral techniques (M = 1.48, SD = 2.53). This indicates a good adherence to both manuals and a small proportion of overlapping therapeutic interventions.

### 3.3 Depression and activation scales

#### 3.3.1 BDI-II

Depression scores in both groups significantly decreased from T0 (M=27.12, SD=11.81) to T3 (M= 14.06, SD=11.69), *t* (797) =15.99, *p* <.001, d= 1.13 and also from T0 to follow up (M= 11.02, SD=15.11), *t* (625) =16.96, *p* <.001, d= 1.28. The results of the comparison tests between the treatment groups are aggregated in **Table 2**. Response rates were calculated according to Hiller and Schindler (49, see **Table 3**). The model that was found to have the best fit based on Akaike Information Criterion (AIC) for BDI-II-score was the one with fixed effects treatment (BA *vs*. CBT), time, and education level, the interaction term treatment x time x education level, and random intercept of patients nested within location (see **Supplementary Table 3 in the Supplementary Material**).

**Table 2:** Means, standard deviations, and d-values with confidence intervals of depression and activation measures.

BA N=174				TAU N=201			
BDI-II	*M*	*SD*	Cohen´s d	BDI-II	*M*	*SD*	Cohen’s d
Pre	25.94	12.65		Pre	27.60	10.56	
Post	15.63	11.97	0.83	Post	16.13	10.61	1.08
			[0.49, 1.18]				[0.71, 1.45]
Follow up (T4)	12.77	14.72	0.97	Follow up (T4)	10.81	14.68	1.33
			[0.61, 1.33]				[0.97, 1.69]
QIDS-C				QIDS-C			
Pre Post*	15.88 7.46	5.62 5.12	1.41 [0.91 1.90]	Pre Post*	15.23 8.16	5.85 5.88	1.23 [0.70, 1.77]
Follow up (T4)	9.08	5.99	1.17	Follow up (T4)	9.05	5.49	1.09
			[0.74, 1.59]				[0.70, 1.49]
Activation				Activation			
Pre	18.99	7.26		Pre	19.12	7.75	
Post	25.02	8.69	0.76	Post	24.86	8.90	0.70
			[0.40, 1.12]				[0.33, 1.06]
Follow up(T4)	20.88	6.25	0.28	Follow up (T4)	21.21	6.48	0.29
			[-0.07,0.63]				[-0.04,0.62]
Avoidance				Avoidance			
Pre	21.88	10.44		Pre	22.31	10.20	
Post	21.37	9.13	0.05	Post	25.76	12.22	0.31
			[-0.29, 0.40]				[-0.04, 0.67]
Follow up (T4)	21.18	7.51	-0.08	Follow up (T4)	22.78	8.87	0.05
			[-0.42,0.27]				[-0.28,0.38]
Work impairment				Work impairment			
Pre	13.14	7.18		Pre	11.97	6.59	
Post	11.78	8.36	0.18	Post	11.14	6.79	0.13
			[-0.17, 0.52]				[-0.23, 0.48]
Follow up (T4)	10.62	5.55	-0.39	Follow up (T4)	11.13	5.49	-0.14
			[-0.74,-0.04]				[-0.47,0.19]
Social impairment				Social impairment			
Pre	13.31	7.22		Pre	12.30	7.38	
Post	9.93	8.34	0.44	Post	12.80	7.33	0.07
			[0.09, 0.78]				[-0.29, 0.42]
Follow up (T4)	9.50	6.08	-0.57	Follow up (T4)	9.13	5.22	-0.49
			[-0.92,-0.21]				[-0.82,-0.16]

M indicates mean. SD indicates standard deviation. d-values are estimates calculated using formulas 4.18 and 4.19 from Borenstein, Hedges, Higgins, & Rothstein (2009). d-values not calculated if unequal variances prevented pooling. Values in square brackets indicate the 95% confidence interval for each d-value The confidence interval is a plausible range of population d-values that could have caused the sample d-value (Cumming, 2014). *Post values of QIDS-C are only from hospital z.

**Table 3:** Response rates at post (T3) of the primary outcome in both treatments.

treatment	response rates	%
BA	non-responder	22.4
	partial responder	23.9
	responder	53.7
CBT	non-responder	20.9
	partial responder	24.9
	responder	54.2

Non-responder means less than 25% of symptom reduction, partial responder: between 49% and 25% of symptom reduction, responder minimum of 50% symptom reduction.

Symptoms are operationalized as sum of BDI-II score (49). BA, Behavioral Activation; CBT, Cognitive Behavioral Therapy.

The estimated variance of patients’ random effects was statistically significant (p <.000), indicating that the within patient variance significantly contributed to the outcome. The ICC for the patients’ random effects nested within a location was 0.48 indicating that 49% of the variance in the outcome was explained by the individual differences between patients and their respective locations.

The effect of time on the outcome was significant in both subgroups, β= -3.78, 95% CI [-4.61; -2.95], *p* <.001 indicating that patients in both treatment groups significantly reduced their symptomatology. Effects of education level (completed vocation training) β= 5.21, 95% CI [0.76;9.65], *p* = .022 and education level (University degree) β= -7. 03, 95% CI [-12.98; -1.07], *p* = .021, were also statistically significant. However, the interaction between time, education level and treatment were not (p=.116). For more detailed information see **Supplementary Table 3 in the Supplementary Material**.

#### 3.3.2 BADS scale

Models that were found to have the best fit based on Akaike Information Criterion (AIC) for BADS Scales (activation, avoidance, social, and work impairments) were the ones with fixed effects treatment (BA *vs*. CBT), time, education level, and random intercept of person nested within a location, see **Supplementary Table 5 in the Supplementary Material**.

Activation was significantly predicted by time β= 1.04, 95% CI [0.35; 1.72], *p*= .003, indicating that patients in both treatment groups increased their activity levels. Social impairment β= - 0.99, 95% CI [-1.57; - -0.41], *p*<.001 significantly decreased over time, whereas work impairment β= - 0.48, 95% CI [-1.03; - 0.07], *p*= .089 did not. Educational level (completed vocational training) had significant effect on avoidance β= 3.89, 95% CI [0.31; 7.47], *p*= .033. The interaction between time, educational level and treatment was not statistically significant for any of the scales, see **Supplementary Table 5 in the Supplementary Material**.

##### 3.3.2.1 QIDS-C

Descriptive analysis revealed that both groups significantly decreased from T0 (M=15.03, SD=5.47) to follow up (M= 8.01, SD=5.91), t(292.44) = 10.43, p <.001, d = 1.21. Additionally, in hospital 2 QIDS-C scores decreased from T0 (M=12.33, SD=4.13) to T3 (M= 7.76, SD=5.40), t(50.15) = 3.79, p <.001, d = 0.91.

Multilevel models: After adjusting for the baseline differences in QIDS scores, no significant impact of treatment on depressive symptoms was found β= - 4.27, 95% CI [-12.94; 4.40], p= .333. The interaction between time, education level and treatment was not statistically significant p=.116. For more detailed information see **Supplementary Table 4 in the Supplementary Material**.

### 3.4 Functioning and participation scale

#### 3.4.1 MINI-ICF-APP

All but few scales significantly changed from pre to follow up. The scales endurance (BA: r = 60 *vs*. CBT: r =53), assertiveness (BA: r= 39 *vs*. CBT: r=63), contact with others (BA: r= 43 *vs*. CBT: r=52), flexibility (BA: r= 44), and group integration (CBT: r= 50) showed increase through higher scores at the end of the treatment (T3). As visual inspection showed increased number of zeros in the data set, we conducted zero-inflated Poisson regression. This analysis confirmed only statistically significant influence of factor time on endurance. Among those who scored minimum 1 point on the MINI-ICF-APP scale, the score changed by a factor of 0.7 from pre to post, and this is statistically significant *p*=.018. For more information see **Tables 4** and **5**.

**Table 4:** Median, Interquartile range (IQR) and effect size r* of Mini-ICF-APP scale.

Time	BA	Median	IQR	r	TAU	Median	IQR	r
	Mini ICF				Mini ICF			
Pre	adherence to regulations	0.00	1.00	0.33	adherence to regulations	0.00	1.00	0.07
Post		0.00	0.00	0.33		0.00	1.00	0.07
Pre	structuring of tasks	1.00	1.00	0.22	structuring of tasks	1.00	2.00	0.32
Post		0.00	1.50	0.22		0.00	1.00	0.32
Pre	flexibility	2.00	2.00	0.44	flexibility	1.00	1.00	0.30
Post		1.00	1.50	0.44		1.00	2.00	0.30
Pre	applying expertise	0.00	1.00	0.20	applying expertise	0.00	1.00	0.08
Post		0.00	1.00	0.20		0.00	1.00	0.08
Pre	competence to judge and decide	1.00	1.00	0.36	competence to judge and decide	1.00	1.00	0.19
Post		1.00	1.00	0.36		0.00	1.00	0.19
Pre	endurance	2.00	1.00	0.60	endurance	2.00	1.00	0.53
Post		1.00	2.00	0.60		1.00	2.00	0.53
Pre	assertiveness	2.00	2.00	0.39	assertiveness	2.00	2.00	0.63
Post		2.00	1.00	0.39		1.00	1.00	0.63
Pre	contact with others	2.00	1.00	0.43	contact with others	2.00	1.00	0.52
Post		1.00	2.00	0.43		1.00	2.00	0.52
Pre	group integration	0.00	1.00	0.16	group integration	1.00	2.00	0.50
Post		0.00	1.00	0.16		1.00	1.00	0.50
Pre	intimate relationships	0.00	1.00	0.08	intimate relationships	0.00	1.00	0.30
Post		0.00	1.00	0.08		0.00	1.00	0.30
Pre	non-work activities	0.00	1.00	0.38	non-work activities	0.00	1.00	0.26
Post		0.00	1.00	0.38		0.00	1.00	0.26
Pre	self-care	0.00	1.00	0.34	self-care	0.00	1.00	0.39
Post		0.00	0.00	0.34		0.00	0.00	0.39
Pre	mobility	0.00	0.00	0.29	mobility	0.00	0.00	0.11
Post		0.00	0.00	0.29		0.00	0.00	0.11

*The effect size r is calculated as Z statistic divided by square root of the sample size (Z/√N, 47).

Rating scale: 0=no impairment, 1=subjective impairment, 3=observable impairment, 3=impairment in need for support by thirds,

4=full impairment (the task has to be overtaken by another person).

**Table 5:** Wilcoxon test results of pre to post change: Z-statistics, p values and effect sizes of Mini-ICF-APP scale.

Treatment	Mini-ICF dimensions	Effect size	n1	n2	Z-Statistic	p	p.adj
BA	adherence to regulations	0.33	55	55	105.00	0.04	0.08
TAU		0.07	55	55	41.00	0.90	1.00
BA	structuring of tasks	0.22	55	55	135.00	0.25	0.50
TAU		0.32	55	55	100.50	0.02	0.03
BA	flexibility	0.44	55	55	320.50	0.00	0.01
TAU		0.30	55	55	155.50	0.04	0.09
BA	applying expertise	0.20	55	55	91.00	0.21	0.41
TAU		0.08	55	55	30.00	0.82	1.00
BA	competence to judge and decide	0.36	55	55	166.00	0.02	0.03
TAU		0.19	55	55	111.00	0.25	0.51
BA	endurance	0.60	55	55	357.00	0.00	0.00
TAU		0.53	55	55	474.50	0.00	0.00
BA	assertiveness	0.39	55	55	355.00	0.01	0.01
TAU		0.63	55	55	411.00	0.00	0.00
BA	contact with others	0.43	55	55	303.00	0.00	0.01
TAU		0.52	55	55	296.50	0.00	0.00
BA	group integration	0.16	55	55	75.00	0.14	0.29
TAU		0.50	55	55	262.50	0.00	0.00
BA	intimate relationships	0.08	55	55	44.50	0.68	1.00
TAU		0.30	55	55	60.00	0.08	0.16
BA	non-work activities	0.38	55	55	100.50	0.02	0.03
TAU		0.26	55	55	44.00	0.07	0.13
BA	self-care	0.34	55	55	50.50	0.02	0.03
TAU		0.39	55	55	72.50	0.01	0.01
BA	mobility	0.29	55	55	32.00	0.04	0.08
TAU		0.11	55	55	14.00	0.48	0.97

*The effect size r is calculated as Z statistic divided by square root of the sample size (Z/√N, 47).

## 4 Discussion

In the present quasi randomized-controlled study, the effects of behavioral activation in a group setting versus cognitive-behavioral group therapy were compared as nested treatment components within a psychosomatic rehabilitation treatment in Germany. Given the large sample and the robust statistical analyses, the study significantly contributes to the rather scarce pool of literature on inpatient group psychotherapy. In line with Folke, Hursti (9), it shows that inpatient psychotherapy *is* beneficial for the treatment of depression, which is of relevant knowledge as it refers to a clientele for which treatment success was not deemed promising in the outpatient setting (e.g., multi-morbid, belongs to marginalized social groups, or is chronically ill).

Our results showed large within-group effect sizes in the decline of depressive symptoms in both treatment groups of about d = 1.24 at follow-up. Furthermore, there were highly favorable response and remission rates in CBT and BA (53.7% and 54.2%, respectively). These effects are among the largest when compared to previous reports on depression treatment in rehabilitation (50) and these results clearly demonstrate the effectiveness of depression treatment within the context of psychosomatic rehabilitation. Compared to outpatient treatment, inpatient treatment is of higher intensity, but also more time-condensed in terms of the duration of stay, which is limited to 5 weeks and can be prolonged to up to 7 weeks. Our results demonstrate that such short-term inpatient psychotherapy programs can be highly effective.

With regards to the comparison of BA *vs*. CBT, no differences were found between both treatments, neither in the change of depressive symptoms nor in the change of any other *main outcomes*. For example, activity levels increased throughout the stay, independent of the form of group treatment applied. These findings are in line with a recent meta-analysis, in which BA compared to an active control group yielded only marginal effect sizes in reduction of depression symptoms as well as increase in activation (51). In line with previous research (52) both treatment modalities showed significant improvement in measures of functional impairment ICF, which, however, did not significantly differ between treatments. This finding does not surprise, as psychosomatic rehabilitations in Germany are packaged treatments that are fine-tuned towards individual assessment and rehabilitation of functional impairments. Differences between BA and CBT regarding ICF were highly improbable, and we did not expect them. Furthermore, patients’ level of education did not moderate the outcome variables. Contrary to our hypothesis, patients with a low education level did not benefit *more* than other patients from the BA component. However, patients with low education showed higher avoidance rates, and social as well as work-related impairments. Interestingly, these findings did not correlate with depression scores as we did not find education level to significantly predict either of the depression measures. Contrary to previous research (53–55), patients with lower education status did not appear to be more depressed. However, psychosomatic rehabilitation clinics not only offer inpatient psychotherapeutic treatment, but they also conduct psychosocial assessment. The results of psychosocial assessment can then lead to measures for further outpatient occupational rehabilitation or even early retirement. Since persons with lower education level (LEL) tend to work in occupations with higher exposure to risks and tend to discharge from work life earlier (54), the above-mentioned results could mirror the fact that for persons with LEL, psychosomatic inpatient psychotherapeutic treatment is as important as psychosocial assessment and occupational therapy.

Overall, the findings suggest that using BA in the context of psychosomatic rehabilitation does not have any add-on effects on treatment outcome. Neither is it inferior to CBT in the present sample. Hence, the evidence of the present study leaves it open which of the two approaches should be preferred in treatment. As both have the same average effects, therapists as well as patients can freely decide which of the two approaches is more convenient and convincing for them. Even if these “weaker” reasons for choosing a treatment approach may not moderate the treatment outcome, they should not be neglected as they are central for what is perceived as a “quality treatment” from the patients’ perspective (56). For many patients, it is of importance how complex, feasible, cost-extensive, comprehensible, fast etc. a treatment is, independent of the outcome (56). Against the backdrop of the advances in personalized medicine, research on psychotherapy has started examining potential of patient-centered treatment. Making a larger variety of treatment modalities available, that can be then tailored towards individual patient’s needs, could lead to better outcomes in the future (57–60). Such development will mirror multi-optional treatment in other medical fields, where patients can e.g., express their wish for or against adjunctive pharmacotherapy. Accommodating individuals’ preferences for *psychotherapy type* could further catalyze the treatment due to a better fit between patient and intervention(s).

At this point, we would like to elaborate on the differences between inpatient psychosomatic rehabilitation and outpatient psychotherapy in Germany. Public insurance companies in Germany reimburse outpatient psychotherapeutic treatment (behavioral therapy), independent of the clinical diagnosis, with up to 60 sessions per case. However, in the practice, the median therapy length is 15,9 months (SD=0.64) and the median number of sessions is 40 (SD= 2 sessions) sessions (1). Since the therapeutic sessions occur on average 1x week, interventions can be precisely tailored towards the demands of the person´s natural environment. On the other hand, inpatient psychosomatic rehabilitation lasts 5 weeks, occasionally up to 7 weeks. This is a very condensed and intense therapeutic treatment package. In particular, it can be very effective for patients that would not be able to make a progress when treated in outpatient setting e.g. due to the severity of the symptoms (2). The challenge than is the transfer and application of newly acquired competencies and skills in the broader contexts of everyday life (e.g. at home, at work etc., 2).

In this respect, it must be emphasized that, behavioral activation is an especially simple and parsimonious method, that is easier to understand for cognitively impaired patients (61). Moreover, it does not require sophisticated therapeutic skills that take years of training (for the therapists of this study applying BA was new). The present study demonstrates that it can be easily integrated into an established and complex treatment program without any negative consequences for the overall effectiveness.

## 5 Limitations

Although specifically targeted, the inpatient setting of this study limits the interpretation of the results. All patients participated in an extensive inpatient treatment program of approximately 25 hrs/week (e.g., occupational therapy, sports, etc.), of which group therapy lasted only 180 min/week. Hence, we could not dismantle the effects of the group therapy modality by itself. Our results should rather be interpreted as a comparison of two treatment packages of which one includes BA and the other CBT. Future studies should direct their attention towards this aspect. Also, the pre-registered sample size of N= 500 could not be achieved due to restrictions of the COVID-19 pandemic. Since we *did not* conceptualize this study as an equivalence trial, we did not set a margin to which we would compare the difference in treatment effects, hence we cannot conclude whether these two methods (BA *vs*. CBT) are equivalent. However, visual inspection of the mean scores showed no substantial differences between the treatment methods, and CIs overlap to a great extent, which is in line with previous research (7). Due to the design of the study, we cannot rule out other factors that might have played a role, such as other treatments (e.g. physical exercise 62), medication, and social environment of the hospital (63). As group therapy is only one aspect of the treatment package within inpatient care, a substantially larger sample or a completely different design (e.g., one arm of the study without any group treatment) would be necessary to detect a possibly small effect. Given the great overlap of the CIs, future researcher should also target the comparison between BA and CBT in terms of an equivalence study.

A further limitation is the number of videotaped sessions of the hospital 1 (8%) that could be used for the adherence check. We chose to follow the well-researched procedure suggested by Dennhag, Gibbons (48). Due to its strictness (e.g., only group sessions led by one therapist, etc.), a large number of group sessions had to be excluded. Considering the context in which this procedure was developed, outpatient addiction treatment in one-to-one setting, it is understandable that such rules have to be followed. However, in inpatient setting of psychosomatic rehabilitation, substitutes lead sessions that would be canceled in outpatient setting. Hence, groups are bound to experience more than one therapist. On the other hand, we can speculate that due to close proximity of team members, and regular meetings such as supervisions, head physician’s weekly rounds etc., adherence can be monitored and maintained better than in an outpatient setting despite sessions being substituted. Since there are no studies focusing on the number of sessions per therapist to check adherence of *inpatient group therapies*, we decided to choose a rather conservative rule suggested above. However, this is a research gap of clinical relevance that future studies should aim at.

Despite our great effort in contacting patients multiple times by multiple means (e.g. by phone, E-mail, mail), we could not prevent high dropout rates at follow up. This is in line with previous research conducted in inpatient settings. Particularly in psychosomatic rehabilitation, response rates have been found to be very low (50). Nevertheless, this reduces the chance to detect differences between the treatments. As the allocation was quasi-randomized, however, neither the researchers nor the patients could influence drop-out rates in any systematic way. Also, limited ethnical and cultural variability of the sample prevents this study from generalizing the results to other ethnical and cultural contexts. Additionally, use of self-reports for testing the main criteria poses a limitation of this study as there are typical risks of biases (e.g., response bias) inherent in the self-report approach. Nevertheless, it has economical and practical advantages for standard use in the inpatient setting and the expert rating measure (QIDS-C) that was conducted at follow up (T4) mirrored the results on the self-report level. Having to interview patients multiple times throughout the study might have additionally reduced the number of participants willing to partake.

Summarizing, this large data set demonstrates behavioral activation to provide a good alternative to cognitive-behavioral therapy in groups especially in fields with a chronically ill, multi-morbid clientele.

## Data availability statement

The original contributions presented in the study are included in the article/**Supplementary Materials**. Further inquiries can be directed to the corresponding author.

## Ethics statement

The studies involving humans were approved by the local ethics committee of the Technische Universität Dresden (Germany; EK 327082018). The studies were conducted in accordance with the local legislation and institutional requirements. The participants provided their written informed consent to participate in this study.

## Author contributions

UM and TS collected and participated in data preparation. TS overseen and conducted the manual adherence measurement. UM wrote the code and analyzed the data. VK and JH, as senior authors, together with UM conceptualized the study and wrote the manuscript. All authors extensively reviewed the manuscript, supplementary materials, and all tables and graphs. All authors contributed to the article and approved the submitted version.
