# Supplementary material for: Behavioral activation for depression in groups embedded in psychosomatic rehabilitation inpatient treatment: a quasi-randomized controlled study
Source: Front Psychiatry. 2024 Apr 25;15:1229380. doi: 10.3389/fpsyt.2024.1229380 (PMC11079813; doi:10.3389/fpsyt.2024.1229380)
Supplement: Supplementary file 1 [file DataSheet_1.docx]

*Statistics*

Data prepraration and statistical analyses were carried out with RStudio (Version 1.3.1093) with R 4.0.3. and additional packages *psych* (Revelle, 2020), *car* (Fox and Weisberg, 2019), *tidyverse (1), haven (2), foreign (3), readxl (4), eeptools (5), lubridate (6), naniar (7), janitor (8), lme4 (9), lmerTest* (10)*, effects (11), datatable (12)*, *_sjPlot (13), apaTables (14)*  *.*

In order to account for dropouts without assuming that the first measurement was stable (i.e., the last observation carried forward assumption), we used a mixed effects models approach with full information maximum likelihood estimation. Furthermore, mixed effect models are able to accommodate missing data and integrate time-varying factors (15). In particular, linear mixed models are also robust to violations of distributional assumptions (16).

A)

First, sum of scores for BDI-II were calculated and then log transformed to conform to normality. To model the development of BDI-II scores between the two treatments linear multi-level model was used. Fixed covariates were *treatment*, *time* and *sex* (measurement points: Pre, T1, T2, T3). To incorporate the dependency among observations within a person, random intercept person was included. If possible, random intercept therapist/group/location would be included. The interaction term was *treatment* and *time*. We used the log likelihood test and the AIC criterion to determine whether the inclusion of each term improved the model fit.

B)

First, sum of scores for BDI-II were calculated and then log transformed to conform to normality. To model the development of BDI-II scores between the two treatments and to incorporate linear multi-level model was used. Fixed covariates were *treatment*, *time* and *sex* (measurement points: Pre, T1, T2). To incorporate the dependency among observations within a person, random intercept person was included. If possible, random intercept therapist/group/location would be included. The interaction term was *treatment* and *time*. We used the log likelihood test and the AIC criterion to determine whether the inclusion of each term improved the model fit.

To analyze the BADS, following steps were conducted. Scales *activation, avoidance, social and work impairement* were calculated with the *scoreItems* function from the psych package. See vignette of the package for more information. Each scale was quantified as a sum of items of BADS-Scales for each patient. Linear mixed effects regression was than estimated. Fixed effects were *treatment*, *time* and *sex* (measurement points Pre, T1, T2, T3). The interaction term was *treatment* and *time*. Random intercept for person was included to model the dependency within a person. If possible, random intercept therapist/group would be included.

*Depression Version B*

a)

First, BDI-II measured at T1 was included as a predictor of the outcome (BDI-Post).

The model that was found to have the best fit based on Akaike Information Criterion (AIC) for BDI-II-score was the one with fixed effects treatment (BA vs. TAU), location and random intercept of group.

The estimated variance of groups’ random effects was significant (p <.000), indicating that the within patient variance significantly contributed to the outcome. The ICC for the groups’ random effect was 26% indicating that 26 % of the outcome was explained by the individual differences between groups. Neither treatment form nor location significantly contributed to the outcome. BDI-T1 was a significant predictor of the outcome β= 0.67, 95% CI [0.63; 0.71], *p*< .001, d= 1.44.

b)

Secondly, BDI-II measured at T2 was included as a predictor of the outcome (BDI-Post).

The model that was found to have the best fit based on Akaike Information Criterion (AIC) for BDI-II-score was the one with fixed effects treatment (BA vs. TAU), location, and random intercept of group.

The estimated variance of groups’ random effects was significant (p <.000), indicating that the within patient variance significantly contributed to the outcome. The ICC for the groups’ random effect was 19% indicating that 19% of the outcome was explained by the individual differences between groups. Neither treatment form nor location significantly contributed to the outcome. BDI-T2 was a significant predictor of the outcome β= 0.82, 95% CI [0.79; 0.85], *p*< .001, d= 0.8.

1. Wickham, al. e. Welcome to the tidyverse. Journal of Open Source

Software

. 2019;4(43).

2. Wickham H, Miller E. haven: Import and Export 'SPSS','Stata' and 'SAS' Files

. R package version 2.3.1. ed2020.

3. R Core Development Team. foreign: Read Data Stored by 'Minitab', 'S', 'SAS', 'SPSS', 'Stata', 'Systat', 'Weka', 'dBase', .... R package version 0.8-80 ed2020.

4. Wickham H, Bryan J. readxl: Read Excel Files. R package version 1.3.1 ed2019.

5. Knowles JE. eeptools: Convenience Functions for Education Data. R package version 1.2.4. ed2020.

6. Grolemund G, Wickham H. Dates and Times Made Easy with lubridate. 2011. 2011;40(3):25.

7. Tierney N, Di Cook, McBain M, Fay C. naniar: Data Structures, Summaries, and Visualisations for Missing Data. R package version 0.6.0 ed2020.

8. Firke S. janitor: Simple Tools for Examining and Cleaning Dirty Data. R package version 2.0.1. ed2020.

9. Bates D, Mächler M, Bolker B, Walker S. Fitting Linear Mixed-Effects Models Using lme4. 2015. 2015;67(1):48.

10. Kuznetsova A, Brockhoff PB, Christensen RHB. lmerTest Package: Tests in Linear Mixed Effects Models. 2017. 2017;82(13):26.

11. Fox J, Hong J. Effect Displays in R for Multinomial and Proportional-Odds Logit Models: Extensions to the effects Package. Journal of Statistical Software. 2009;32(1):1-24.

12. Dowle M, Srinivasan A. data.table: Extension of `data.frame`. R package version 1.13.4 ed2020.

13. Lüdecke D. _sjPlot: Data Visualization for Statistics in Social

Science_. R package version 2.8.12 ed2022.

14. Stanley D. _apaTables: Create American Psychological Association

(APA) Style Tables_. R package version 2.0.8 ed2021.

15. Tasca GA, Gallop R. Multilevel modeling of longitudinal data for psychotherapy researchers: I. The basics. Psychotherapy Research. 2009;19(4-5):429-37.

16. Schielzeth H, Dingemanse NJ, Nakagawa S, Westneat DF, Allegue H, Teplitsky C, et al. Robustness of linear mixed-effects models to violations of distributional assumptions. Methods in Ecology and Evolution. 2020;11(9):1141-52.
