# Supplementary material for: Behavioral activation for depression in groups embedded in psychosomatic rehabilitation inpatient treatment: a quasi-randomized controlled study
Source: Front Psychiatry. 2024 Apr 25;15:1229380. doi: 10.3389/fpsyt.2024.1229380 (PMC11079813; doi:10.3389/fpsyt.2024.1229380)
Supplement: Supplementary file 2 [file Image_1.pdf]

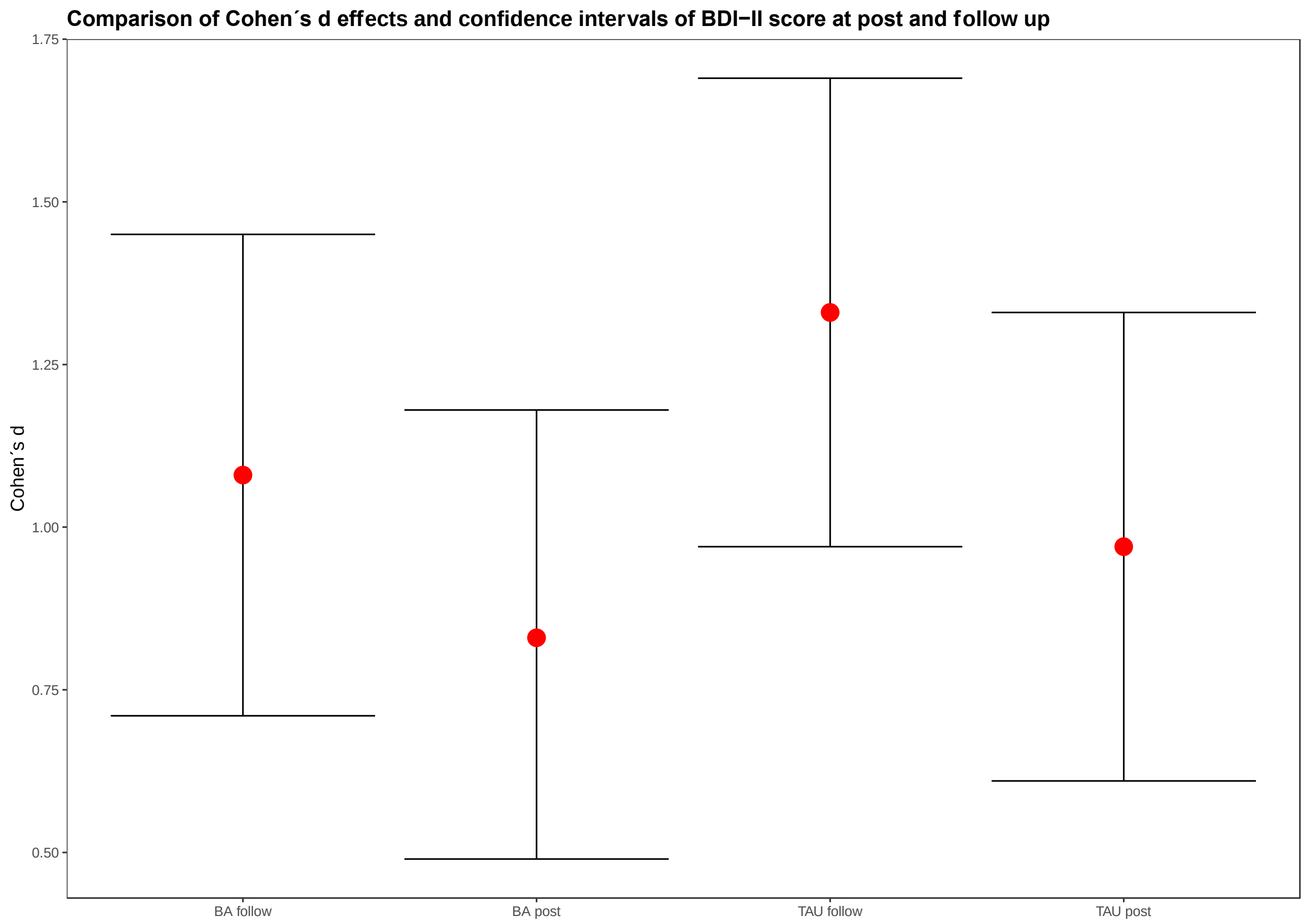

Supplementary Figure 1. Red dots depict Cohen's effect sizes and lines represent confidence intervals. CBT= cognitive behavioral therapy, BA= behavioral activation.
