# Supplementary material for: Behavioral activation for depression in groups embedded in psychosomatic rehabilitation inpatient treatment: a quasi-randomized controlled study
Source: Front Psychiatry. 2024 Apr 25;15:1229380. doi: 10.3389/fpsyt.2024.1229380 (PMC11079813; doi:10.3389/fpsyt.2024.1229380)
Supplement: Supplementary file 3 [file Image_2.pdf]

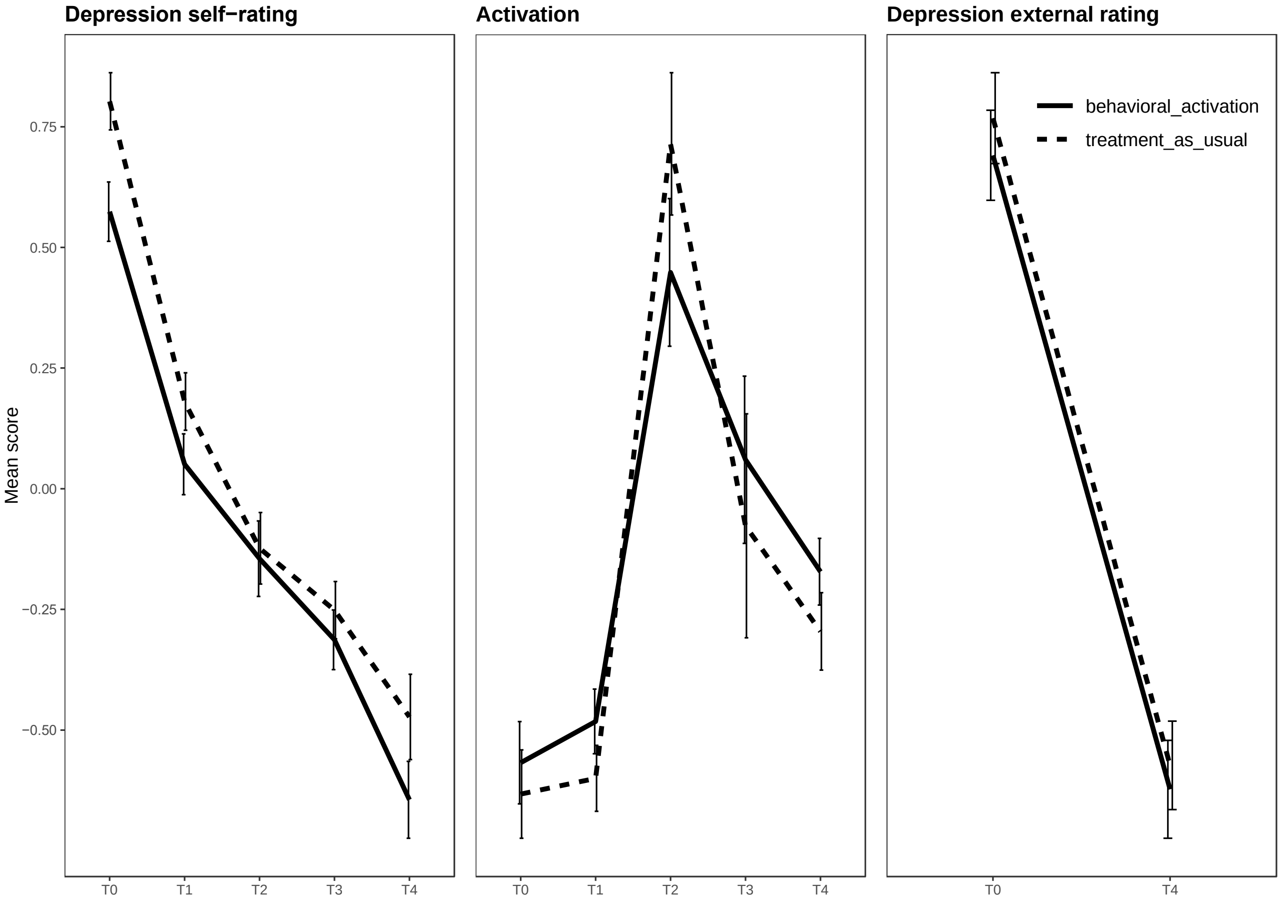

Supplementary Figure 2. z-transformed mean scores of self-rating BDI-II scale, BADS activation scale (throughout the treatment), and external rating QIDS-C (pre= T0 to follow up= T4
