# Supplementary material for: Behavioral activation for depression in groups embedded in psychosomatic rehabilitation inpatient treatment: a quasi-randomized controlled study
Source: Front Psychiatry. 2024 Apr 25;15:1229380. doi: 10.3389/fpsyt.2024.1229380 (PMC11079813; doi:10.3389/fpsyt.2024.1229380)
Supplement: Supplementary file 4 [file Table_1.docx]

Supplementary Table 1: Selected socio-demographic data

on non-completers of follow up data collection.

| **Characteristic** | **BA**  N = 73*^1^* | **TAU**,  N = 72*^1^* | **p-value***^2^* |
| --- | --- | --- | --- |
| BDI pre | 21 (13, 27) | 26 (15, 34) | 0.087 |
| sex |  |  | 0.2 |
| Female | 56 (77%) | 49 (68%) |  |
| age | 54 (48, 58) | 52 (45, 57) | 0.5 |
| education level |  |  | 0.012 |
| special needs school | 0 (0%) | 0 (0%) |  |
| secondary school certificate | 4 (5.5%) | 2 (2.8%) |  |
| compl.vocational training | 35 (48%) | 51 (71%) |  |
| A-levels | 17 (23%) | 14 (19%) |  |
| University degree | 17 (23%) | 5 (6.9%) |  |
| f1 | 4 (5.5%) | 4 (5.6%) | >0.9 |
| f40_f41 | 37 (51%) | 33 (46%) | 0.6 |
| Missing | 0 | 1 |  |
| f42 | 5 (6.8%) | 1 (1.4%) | 0.2 |
| ptbs | 7 (9.6%) | 4 (5.6%) | 0.4 |
| f5 | 4 (5.5%) | 1 (1.4%) | 0.4 |
| dysthymia | 17 (23%) | 17 (24%) | >0.9 |
| *^1^*Median (IQR); n (%)  *^2^*Wilcoxon rank sum test; Pearson's Chi-squared test; Fisher's exact test | | | |
