# Supplementary material for: Behavioral activation for depression in groups embedded in psychosomatic rehabilitation inpatient treatment: a quasi-randomized controlled study
Source: Front Psychiatry. 2024 Apr 25;15:1229380. doi: 10.3389/fpsyt.2024.1229380 (PMC11079813; doi:10.3389/fpsyt.2024.1229380)
Supplement: Supplementary file 5 [file Table_2.docx]

Supplementary Table 2: Selected socio-demographic data on

completers of follow up data collection.

| **Characteristic** | **BA**  N = 101*^1^* | **TAU**  N = 129*^1^* | **p-value***^2^* |
| --- | --- | --- | --- |
| BDI pre | 27 (19, 36) | 31 (22, 38) | 0.11 |
| sex |  |  | 0.8 |
| Female | 79 (78%) | 103 (80%) |  |
| age | 54 (47, 58) | 54 (49, 60) | 0.2 |
| edlev |  |  | 0.5 |
| special needs school | 0 (0%) | 0 (0%) |  |
| secondary school certificate | 2 (2.0%) | 6 (4.7%) |  |
| compl.vocational training | 63 (62%) | 78 (60%) |  |
| A-levels | 28 (28%) | 30 (23%) |  |
| University degree | 8 (7.9%) | 15 (12%) |  |
| f1 | 4 (4.0%) | 6 (4.7%) | >0.9 |
| f40_f41 | 65 (64%) | 69 (53%) | 0.10 |
| f42 | 4 (4.0%) | 7 (5.4%) | 0.8 |
| ptbs | 13 (13%) | 13 (10%) | 0.5 |
| f5 | 0 (0%) | 2 (1.6%) | 0.5 |
| dysthymia | 18 (18%) | 21 (16%) | 0.7 |
| Missing | 1 | 0 |  |
| *^1^*Median (IQR); n (%)  *^2^*Wilcoxon rank sum test; Pearson's Chi-squared test; Fisher's exact test | | | |
