# Supplementary material for: Behavioral activation for depression in groups embedded in psychosomatic rehabilitation inpatient treatment: a quasi-randomized controlled study
Source: Front Psychiatry. 2024 Apr 25;15:1229380. doi: 10.3389/fpsyt.2024.1229380 (PMC11079813; doi:10.3389/fpsyt.2024.1229380)
Supplement: Supplementary file 6 [file Table_3.docx]

Supplementary Table 3:

Multilevel Model of self-rating depression scale (BDI-II).

|  | **Mean BDI-II** | |
| --- | --- | --- |
| *Predictors* | *Estimates (CI)** | *p* |
| Education level  [Compl.vocational training] | 5.21 ^*^ (0.76 – 9.65) | **0.022** |
| Education level  [Secondary school certificate] | 10.72  (-1.00 – 22.45) | 0.073 |
| Education level  [University degree] | -7.03 ^*^ (-12.98 – -1.07) | **0.021** |
| Time | -3.78 ^***^ (-4.61 – -2.95) | **<0.001** |
| Time x Education level  [Compl.vocational training] | -0.66  (-1.66 – 0.33) | 0.192 |
| Time x Education level  [Secondary school certificate] | -0.52  (-3.39 – 2.34) | 0.720 |
| Time x Education level  [University degree] | 1.75 ^**^ (0.44 – 3.06) | **0.009** |
| Treatment  [TAU] | 3.99  (-1.22 – 9.20) | 0.134 |
| Treatment [TAU] x Education level  [Compl.vocational training] | -4.23  (-10.41 – 1.96) | 0.180 |
| Treatment [TAU] x Education level  [Secondary school certificate] | -0.51  (-15.64 – 14.61) | 0.947 |
| Treatment [TAU] x Education level  [University degree] | 1.61  (-7.31 – 10.52) | 0.724 |
| Treatment [TAU] x Time | -0.10  (-1.28 – 1.08) | 0.865 |
| Treatment [TAU] x Time x Education level  Compl.vocational training | 0.46  (-0.94 – 1.85) | 0.520 |
| Treatment [TAU] x Time x Education level  Secondary school certificate | -2.49  (-6.11 – 1.13) | 0.178 |
| Treatment [TAU] x Time x Education level  University degree | -1.26  (-3.24 – 0.71) | 0.210 |
| **Random Effects** | | |
| σ^2^ | 76.72 | |
| τ_00_ _location:ID_ | 71.77 | |
| ICC | 0.48 | |
| N _location_ | 2 | |
| N _ID_ | 365 | |
| Observations | 1742 | |
| Marginal R^2^ / Conditional R^2^ | 0.208 / 0.591 | |
| ** p<0.05   ** p<0.01   *** p<0.001*  Note: σ^2^ represents mean random variance of the model or within group variance.  τ_00_ _ID_ represents between group variance, in this case variance between patients.  ICC = intra-class correlation coefficient or how much variability is between patients.  CI= Confidence intervals, in brackets. | | |
