# Supplementary material for: Behavioral activation for depression in groups embedded in psychosomatic rehabilitation inpatient treatment: a quasi-randomized controlled study
Source: Front Psychiatry. 2024 Apr 25;15:1229380. doi: 10.3389/fpsyt.2024.1229380 (PMC11079813; doi:10.3389/fpsyt.2024.1229380)
Supplement: Supplementary file 7 [file Table_4.docx]

Supplementary Table 4: Multilevel model of depression score:

external rating QIDS-C

|  | **QIDS-C score at follow up** | | | | |
| --- | --- | --- | --- | --- | --- |
| *Predictors* | *Estimates* | *CI* | | *p* | |
| QIDS score at T0 | 0.36 ^***^ | 0.22 – 0.50 | | **<0.001** | |
| Treatment [TAU] | -4.27 | -12.94 – 4.40 | | 0.333 | |
| Education level  [compl. vocational training] | -4.25 | -11.74 – 3.25 | | 0.265 | |
| Education level  [secondary school certificate] | -4.10 | -11.83 – 3.62 | | 0.296 | |
| Education level  [University degree] | -5.27 | -13.41 – 2.87 | | 0.203 | |
| Treatment [TAU]× Education level  [compl. vocational training] | 4.18 | -4.67 – 13.03 | | 0.352 | |
| Treatment [TAU]× Education level  [secondary school certificate] | 4.33 | -4.82 – 13.48 | | 0.352 | |
| Treatment [TAU]× Education level  [University degree] | 4.48 | -5.31 – 14.28 | | 0.368 | |
| σ^2^ | 26.86 | |  | |  |
| τ_00_ | 0.81 _location:group id_ | |  | |  |
| ICC | 0.03 | |  | |  |
| N | 2 _location_ | |  | |  |
|  | 67 _group_id_ | |  | |  |
| Observations | 211 | |  | |  |
| Marginal R^2^ / Conditional R^2^ | 0.133 / 0.159 | |  | |  |
