# Supplementary material for: Behavioral activation for depression in groups embedded in psychosomatic rehabilitation inpatient treatment: a quasi-randomized controlled study
Source: Front Psychiatry. 2024 Apr 25;15:1229380. doi: 10.3389/fpsyt.2024.1229380 (PMC11079813; doi:10.3389/fpsyt.2024.1229380)
Supplement: Supplementary file 8 [file Table_5.docx]

Supplementary Table 5. Multilevel model of activation scale (BADS).

|  | **Activation** | | | **Avoidance** | | | **Social impairement** | | | **Work impairement** | | |
| --- | --- | --- | --- | --- | --- | --- | --- | --- | --- | --- | --- | --- |
| *Predictors* | *Estimates* | *CI* | *p* | *Estimates* | *CI* | *p* | *Estimates* | *CI* | *p* | *Estimates* | *CI* | *p* |
| Time | 1.04 | 0.35 – 1.72 | **0.003** | 0.53 | -0.33 – 1.39 | 0.227 | -0.99 | -1.57 – -0.41 | **0.001** | -0.48 | -1.03 – 0.07 | 0.089 |
| Treatment [TAU] | 0.95 | -2.43 – 4.32 | 0.582 | 1.93 | -2.26 – 6.12 | 0.367 | -1.09 | -4.32 – 2.14 | 0.508 | -2.10 | -5.20 – 1.01 | 0.186 |
| Education level  [compl. vocational training] | -1.03 | -3.91 – 1.86 | 0.486 | 3.89 | 0.31 – 7.47 | **0.033** | 1.43 | -1.32 – 4.17 | 0.309 | 0.18 | -2.46 – 2.82 | 0.893 |
| Education level  [secondary school certificate] | -6.45 | -14.84 – 1.93 | 0.131 | 2.16 | -8.25 – 12.56 | 0.684 | 6.21 | -1.65 – 14.08 | 0.122 | 4.48 | -3.10 – 12.05 | 0.246 |
| Education level [University degree] | 1.44 | -2.37 – 5.25 | 0.458 | -1.04 | -5.76 – 3.69 | 0.667 | -3.06 | -6.63 – 0.51 | 0.093 | -2.37 | -5.81 – 1.07 | 0.176 |
| Time × Treatment [TAU] | -0.53 | -1.50 – 0.45 | 0.288 | -0.21 | -1.43 – 1.01 | 0.740 | 0.40 | -0.42 – 1.23 | 0.341 | 0.46 | -0.32 – 1.25 | 0.246 |
| Time × Education level  [compl. vocational training] | -0.15 | -0.97 – 0.67 | 0.716 | -0.71 | -1.74 – 0.32 | 0.176 | -0.28 | -0.97 – 0.42 | 0.433 | -0.19 | -0.85 – 0.47 | 0.564 |
| Time × Education level  [secondary school certificate] | 1.21 | -1.60 – 4.02 | 0.398 | 0.20 | -3.32 – 3.72 | 0.911 | -1.09 | -3.48 – 1.29 | 0.369 | -0.03 | -2.30 – 2.24 | 0.978 |
| Time × Education level [University degree] | -0.60 | -1.65 – 0.45 | 0.261 | 0.35 | -0.97 – 1.66 | 0.607 | 0.71 | -0.17 – 1.59 | 0.115 | 0.13 | -0.71 – 0.97 | 0.764 |
| Treatment [TAU] ×  Education level  [compl.vocational training] | -1.52 | -5.53 – 2.48 | 0.456 | -3.28 | -8.25 – 1.70 | 0.197 | 0.83 | -2.99 – 4.65 | 0.671 | 2.09 | -1.59 – 5.78 | 0.264 |
| Treatment [TAU] ×  Education level  [secondary school certificate] | 6.65 | -3.77 – 17.07 | 0.211 | 8.74 | -4.20 – 21.68 | 0.185 | 6.19 | -3.67 – 16.05 | 0.218 | 7.55 | -1.94 – 17.04 | 0.119 |
| Treatment [TAU] ×  Education level  [University degree] | -1.03 | -6.75 – 4.70 | 0.725 | 3.31 | -3.80 – 10.42 | 0.362 | 4.29 | -1.06 – 9.64 | 0.116 | 3.40 | -1.75 – 8.55 | 0.196 |
| Time × Treatment [TAU] × Education level [compl. vocational training] | 0.52 | -0.63 – 1.67 | 0.373 | 0.86 | -0.58 – 2.30 | 0.241 | 0.05 | -0.93 – 1.02 | 0.925 | -0.13 | -1.05 – 0.79 | 0.783 |
| Time × Treatment [TAU] × Education level [secondary school certificate] | -1.37 | -4.73 – 1.99 | 0.423 | -2.07 | -6.28 – 2.13 | 0.334 | -1.37 | -4.21 – 1.48 | 0.345 | -2.08 | -4.79 – 0.62 | 0.131 |
| Time × Treatment [TAU] × Education level [University degree] | 0.90 | -0.67 – 2.48 | 0.260 | -1.49 | -3.46 – 0.48 | 0.137 | -1.29 | -2.61 – 0.03 | 0.055 | -0.61 | -1.86 – 0.64 | 0.338 |
| **Random Effects** | | | | | | | | | | | | |
| σ^2^ | 43.09 | | | 67.67 | | | 28.63 | | | 25.80 | | |
| τ_00_ | 16.11 _location:ID_ | | | 23.48 _location:ID_ | | | 17.50 _location:ID_ | | | 17.23 _location:ID_ | | |
| ICC | 0.27 | | | 0.26 | | | 0.38 | | | 0.40 | | |
| N | 2 _location_ | | | 2 _location_ | | | 2 _location_ | | | 2 _location_ | | |
|  | 364 _ID_ | | | 364 _ID_ | | | 362 _ID_ | | | 362 _ID_ | | |
| Observations | 1390 | | | 1390 | | | 1072 | | | 1072 | | |
| Marginal R^2^ / Conditional R^2^ | 0.040 / 0.302 | | | 0.023 / 0.275 | | | 0.086 / 0.433 | | | 0.049 / 0.429 | | |

Note: σ^2^ represents mean random variance of the model or within group variance.

τ_00_ _location:ID_ represents between group variance, in this case variance between groups nested within location.

ICC:intra-class correlation coefficient or how much variability is between patients
