# Supplementary material for: Behavioral activation for depression in groups embedded in psychosomatic rehabilitation inpatient treatment: a quasi-randomized controlled study
Source: Front Psychiatry. 2024 Apr 25;15:1229380. doi: 10.3389/fpsyt.2024.1229380 (PMC11079813; doi:10.3389/fpsyt.2024.1229380)
Supplement: Supplementary file 9 [file Table_6.docx]

| **BA**  Supplementary Table 6. Median, Interquartile range (IQR) and effect of Mini-ICF APP-S scale at hospital 2. |  |  |  |  |  | **TAU** |  |  |  |  |
| --- | --- | --- | --- | --- | --- | --- | --- | --- | --- | --- |
| *Pre* |  |  |  |  |  | *Pre* |  |  |  |  |
| Mini | Median | Max | Min | IQR |  | Mini | Median | Max | Min | IQR |
| adherence to regulations | 3.00 | 5.00 | 1.00 | 2.00 |  | adherence to regulations | 3.00 | 4.00 | 1.00 | 0.75 |
| structuring of tasks | 3.00 | 6.00 | 1.00 | 2.00 |  | structuring of tasks | 3.50 | 4.00 | 1.00 | 1.50 |
| flexibility | 4.00 | 7.00 | 1.00 | 2.00 |  | flexibility | 4.00 | 6.00 | 1.00 | 2.75 |
| applying expertise | 3.00 | 6.00 | 1.00 | 1.00 |  | applying expertise | 3.00 | 6.00 | 1.00 | 1.25 |
| competence to judge and decide | 3.00 | 5.00 | 1.00 | 1.00 |  | competence to judge and decide | 3.50 | 5.00 | 2.00 | 1.50 |
| endurance | 4.00 | 8.00 | 1.00 | 2.00 |  | endurance | 5.00 | 6.00 | 3.00 | 2.25 |
| assertiveness | 4.00 | 8.00 | 1.00 | 2.50 |  | assertiveness | 4.50 | 5.00 | 3.00 | 1.25 |
| contact with others | 5.00 | 8.00 | 3.00 | 3.00 |  | contact with others | 4.00 | 6.00 | 2.00 | 2.50 |
| group integration | 4.00 | 8.00 | 1.00 | 2.00 |  | group integration | 3.50 | 5.00 | 2.00 | 1.50 |
| intimate relationships | 3.00 | 8.00 | 1.00 | 1.50 |  | intimate relationships | 4.00 | 6.00 | 3.00 | 2.25 |
| non-work activities | 3.00 | 8.00 | 1.00 | 0.00 |  | non-work activities | 3.00 | 3.00 | 3.00 | 0.00 |
| self-care | 3.00 | 7.00 | 1.00 | 1.50 |  | self-care | 4.00 | 6.00 | 3.00 | 2.25 |
| mobility | 3.00 | 7.00 | 1.00 | 0.50 |  | mobility | 4.00 | 6.00 | 3.00 | 2.25 |
| *Post* |  |  |  |  |  | *Post* |  |  |  |  |
| Mini | Median | Max | Min | IQR |  | Mini | Median | Max | Min | IQR |
|  |  |  |  |  |  |  |  |  |  |  |
| adherence to regulations | 3.00 | 6.00 | 1.00 | 3.00 |  | adherence to regulations | 3.50 | 4.00 | 1.00 | 1.50 |
| structuring of tasks | 3.00 | 6.00 | 1.00 | 2.00 |  | structuring of tasks | 4.00 | 5.00 | 1.00 | 2.50 |
| flexibility | 3.00 | 7.00 | 1.00 | 2.50 |  | flexibility | 4.00 | 7.00 | 1.00 | 3.00 |
| applying expertise | 3.00 | 6.00 | 1.00 | 1.50 |  | applying expertise | 3.50 | 5.00 | 1.00 | 1.75 |
| competence to judge and decide | 3.00 | 5.00 | 1.00 | 1.50 |  | competence to judge and decide | 3.50 | 5.00 | 2.00 | 1.50 |
| endurance | 3.00 | 8.00 | 1.00 | 2.50 |  | endurance | 4.50 | 6.00 | 3.00 | 1.50 |
| assertiveness | 4.00 | 8.00 | 1.00 | 3.50 |  | assertiveness | 4.00 | 8.00 | 3.00 | 2.75 |
| contact with others | 4.00 | 8.00 | 3.00 | 2.50 |  | contact with others | 3.50 | 6.00 | 2.00 | 1.75 |
| group integration | 3.00 | 8.00 | 1.00 | 2.50 |  | group integration | 4.00 | 6.00 | 3.00 | 2.25 |
| intimate relationships | 3.00 | 8.00 | 1.00 | 1.50 |  | intimate relationships | 3.50 | 6.00 | 3.00 | 1.50 |
| non-work activities | 3.00 | 8.00 | 1.00 | 1.00 |  | non-work activities | 3.00 | 3.00 | 3.00 | 0.00 |
| self-care | 3.00 | 8.00 | 1.00 | 2.50 |  | self-care | 3.50 | 5.00 | 2.00 | 1.50 |
| mobility | 3.00 | 6.00 | 1.00 | 1.50 |  | mobility | 3.00 | 6.00 | 3.00 | 0.75 |
